# Sodium–glucose cotransporter 2 inhibitors as an add-on therapy to insulin for type 1 diabetes mellitus: Meta-analysis of randomized controlled trials

**DOI:** 10.1007/s00592-021-01686-x

**Published:** 2021-03-02

**Authors:** Lunwen Rao, Chenhong Ren, Shan Luo, Chenghu Huang, Xuefeng Li

**Affiliations:** 1 Huangdu Community Health Service Center, Jiading District, Anting TownShanghai, China; 2 Postgraduate Training Basement of Jinzhou Medical University, Taihe Hospital, Hubei University of Medical, Shiyan, China; 3 Department of Endocrinology, Taihe Hospital, Hubei University of Medical, Shiyan, 442000 China; 4 Department of Endocrinology, The People’s Hospital of Bishan District, Bishan, Chongqing, 402760 China

**Keywords:** Sodium–glucose cotransporter 2 inhibitor, Add-on therapy, Type 1 diabetes mellitus, Meta-analysis

## Abstract

**Aims:**

The aim was to systematically review the efficacy and safety of sodium–glucose cotransporter inhibitor (SGLT2i) as an adjunct to insulin at different follow-up durations in randomized, double-blind clinical trials in patients with type 1 diabetes.

**Methods:**

We conducted a search on Medline, Embase, and the Cochrane Library for relevant studies published before May 2020. According to the duration of follow-up, the subgroup analysis included four periods: 1–4, 12–18, 24–26, and 52 weeks. In the five trials included both 24–26 and 52 weeks of follow-up, we compared the efficacy by the placebo-subtracted difference and changes in SGLT2i groups.

**Results:**

Fifteen trials including 7109 participants were analyzed. The combination of SGLT2i and insulin improved hemoglobin A1c (HbA1c), fasting plasma glucose (FPG), daily insulin dose, body weight, and blood pressure, which varied greatly by different follow-ups. Compared with %HbA1c at 24–26 weeks, placebo-subtracted differences and changes in the SGLT2i groups slightly increased. SGLT2i plus insulin treatment showed no difference in the occurrence of urinary tract infections (UTIs), hypoglycemia, or severe hypoglycemia but increased the risk of genital tract infections (GTIs) in a duration-dependent manner. SGLT2i treatment was associated with a significantly higher rate of ketone-related SAEs and diabetic ketoacidosis (DKA) at 52 weeks.

**Conclusion:**

SGLT2i as an add-on therapy to insulin improved glycemic control and body weight and decreased the required dose of insulin without increasing the risk of hypoglycemia. However, after 6 months the benefits of SGLT2is on glycemic control may weaken and the risks of GTIs and DKA increased.

**Supplementary Information:**

The online version contains supplementary material available at (10.1007/s00592-021-01686-x).

## Introduction

Diabetes mellitus (DM) is a systemic disease associated with an increased risk of adverse vascular events. DCCT-EDIC and UKPDS have shown that improved glucose control through an increase in insulin therapy is associated with reductions in the long-term risks of both microvascular and macrovascular events [1, 2]. However, the achievement and maintenance of glycemic targets have proven both difficult and hazardous, especially in type 1 diabetes (T1DM) [3, 4].

Over several decades, the T1DM prevalence has increased, seriously affecting the whole world [5, 6]. Although advances in medical and management technology for T1DM have been made, mortality remains almost unchanged among adults aged 20–44 years [7]. The mainstay of treatment for T1DM is still insulin therapy, with a varying degree of side effects including hypoglycemia and weight gain. Consequently, there is an unmet need for adjunctive treatment plus insulin in T1DM to meet the twin challenges of hyperglycemia and hypoglycemia [8, 9]. Many different oral antidiabetic drugs (OADs) combined with insulin have been approved for type 2 diabetes (T2DM), including metformin, incretin analogs, and sodium–glucose cotransporter (SGLT) 2 inhibitors (SGLT-2is) [10–12]. These drugs can improve insulin resistance and blood glucose levels, reduce the incidence of hypoglycemia, and manage body weight. Unfortunately, unlike for patients with T2DM, the options for those with T1DM are limited [8, 9, 13].

SGLT-2is are a novel class of antidiabetic agents, such as canagliflozin, dapagliflozin, empagliflozin, ipragliflozin, and tofogliflozin. SGLT-2is reduce glucose reabsorption at the proximal nephron, leading to increased glucose excretion through a mechanism that is independent of insulin [14–16]. Recently, SGLT-2is have become an attractive therapeutic proposition for diabetes patients due to their additional beneficial biological effects other than glycemic control, including decreased blood pressure, body weight loss, and reduced cardiovascular mortality in patients with T2DM [14, 15, 17, 18]. The recent publication of several large randomized controlled trials (RCTs) reported the benefits of SGLT2 inhibitors on the decrease in hemoglobin A1c (HbA1c), fasting plasma glucose (FPG) levels in T1DM with insulin therapy [19–34]. Nevertheless, questions remain regarding the long-term efficacy and safety of SGLT2 inhibitors as add-ons to insulin in the treatment of T1DM. There is no study to systematically review the efficacy and safety of the combination of SGLT2 inhibitors and insulin compared with insulin monotherapy in the different treatment periods. Therefore, the aim of the present study was to evaluate the relative effectiveness and safety of this important therapeutic course by a meta-analysis of randomized controlled trials in T1DM with insulin therapy.

## Methods

### Materials and methods

This study is a systematic review and meta-analysis assessing the duration of effects of SGLT2is for adjunctive treatment of T1DM. The extensive searches were carried out in PubMed, EMBASE, and the Cochrane Central Register of Controlled Trials (CENTRAL) through May 2020, using both Medical Subject Heading (MeSH) and free text terms. We also searched ClinicalTrials.gov to identify additional relevant trials. This meta-analysis was performed by following Cochrane Collaboration guidelines and is reported in accordance with the preferred reporting items for systematic reviews and meta-analyses (PRISMA) statement.

### Primary and secondary endpoints

Studies included in the meta-analyses are randomized controlled trials (RCTs) that evaluated the efficacy, safety, and tolerability of SGLT2is as add-ons to insulin compared with placebo combination. Participants were T1DM patients having inadequate control of disease by insulin therapy (multiple daily insulin injections or insulin pump). Randomized trials that fulfilled the following criteria were eligible: (1) comparison of SGLT2-i therapy with placebo in adult patients (≥ 18 years old) with type 1 diabetes, and (2) reporting efficacy and safety outcomes of interest. Studies were excluded if other aspects of treatment were targeted, if the design was not double-blind (e.g., open-label or crossover). Studies of children and observational studies were also ineligible.

Primary outcome measures of interest were the changes from baseline in percent HbA1c, FPG levels, and body weight. Secondary endpoints were the changes from baseline in systolic blood pressure (SBP), diastolic blood pressure (DBP), and daily insulin doses (basal, bolus, and total insulin doses). Safety endpoints were the incidence of hypoglycemia (including severe hypoglycemia), genital tract infections, urinary tract infections, and ketoacidosis. The definitions of severe hypoglycemia and diabetic ketoacidosis (DKA) were based on those of a previous meta-analysis [35].

### Data extraction, synthesis, and statistical analysis

Data extraction was carried out by two reviewers (Lunwen Rao and Chenhong Ren) independently by adapting a standardized procedure. Data pertaining to the participants’ demographic and pathological characteristics, intervention design, trial eligibility criteria, outcome measures, and outcomes were extracted from the selected research articles. According to the duration of follow-up, the subgroup analysis from the consolidation of ranges included four periods: 1–4, 12–18, 24–26, and 52 weeks. Changes from baseline in the endpoints were either extracted directly from the respective research articles if provided or calculated from the baseline values and experimental values noted. Quality assessment of the RCTs included in this meta-analysis was carried out by using the Cochrane risk of bias tool [36]. For continuous outcomes, mean differences and 95% confidence intervals were calculated by an inverse variance random-effects model. For dichotomous outcomes, risk ratios and 95% confidence intervals were calculated by the random-effects Mantel–Haenszel approach [37]. Data and analysis module of RevMan (version 5.2; Cochrane Collaboration) was used for the meta-analyses. Between-studies (heterogeneity) was tested by I^2^ statistics, and a p value of less than 0.05 was considered statistically significant.

### Results

**Fig. 1:**
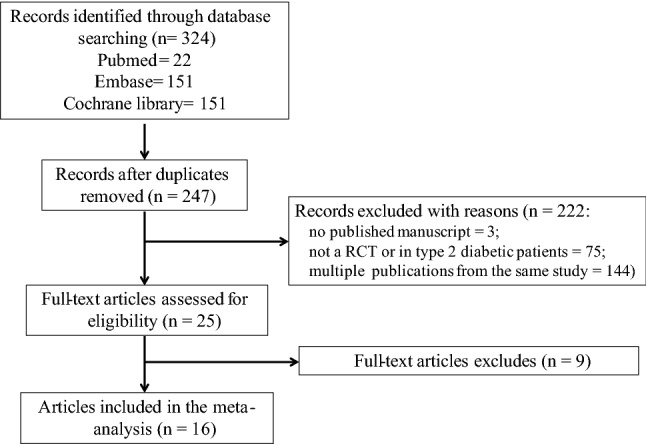
Flow diagram of study selection

A total of 324 articles were retrieved initially utilizing the search strategy (Fig. 1). After the removal of 77 duplicate articles, 247 articles remained for title and abstract screening. A total of 222 articles were ruled out on the basis of titles and abstracts. Nine articles were included for full-text screening. There were two studies in which the risk of bias could not be judged due to inadequate information [38, 39]. Fifteen randomized placebo-controlled trials (*n* = 7,109 patients) satisfied the inclusion criteria. We included five trials of sotagliflozin, four trials of empagliflozin, three trials of dapagliflozin, two trials of ipragliflozin, and one trial of canagliflozin. The baseline characteristics and results were obtained in trials in Tables S1 and S2 of Supplementary Materials. The mean hemoglobin A1C (HbA1c), total insulin dose, and body mass index (BMI) were 8.0–8.5%, 0.6–0.7 units/kg/day, and 23–29 kg/m^2^, respectively. These factors were balanced between groups.

The duration of follow-up varied widely, including four trials at 1–4 weeks, two trials at 12–16 weeks, eight trials at 24–26 weeks, and five trials at 52 weeks. And the five trials included both 24–26 and 52 weeks of follow-up [23, 25, 29, 31, 32, 34]. Definitions of hypoglycemia were similar in all trials and followed the American Diabetes Association criteria. Not all studies reported all the outcomes. In general, the majority of the domains for the seventeen studies were considered to have a high quality and a low risk of bias. Risks of bias assessments are included in Figures S1 and S2.

## Efficacy of SGLT-2i intervention as add-ons

### Pooled analysis

Not all studies reported all the outcomes (Table 1). There was no significant heterogeneity between studies in terms of HbA1c, fasting plasma glucose (FPG), daily insulin dose (total, basal, and bolus), and seated blood pressure (systolic and diastolic) (*P* > 0.05, respectively). Large heterogeneity was noted between studies only in terms of body weight (*P* < 0.001, *I*^2^ = 82%). However, the combination of SGLT2is and insulin treatment markedly reduced HbA1c, fasting plasma glucose (FPG), body weight, daily insulin dose (total, basal, and bolus), and seated blood pressure (systolic and diastolic) (*P* < 0.001).

**Table 1:** Summary of results of efficacy changes comparing SGLT2i with placebo in T1DM patients with insulin treatment

Comparison		No. of studies	Participants (SGLT2i/placebo)	Overall effect		Heterogeneity		
Outcome	Subgroup			Pooled results (95% CI)	*P*	Tau^2^	*I*^2^, %	*P*
%HbA1c	Overall	13	4401/2455	− 0.39 [ − 0.43, − 0.35]	< 0.00001	0.00	0	0.86
	Canagliflozin	1	234/117	− 0.27 [ − 0.52, − 0.02]	0.03	NP	NP	NP
	Dapagliflozin	2	1007/500	− 0.41[ − 0.50, − 0.32]	< 0.00001	0.00	0	0.45
	Empagliflozin	4	1258/519	− 0.38[ − 0.45, − 0.31]	< 0.00001	0.00	0	0.70
	Ipragliflozin	1	115/59	− 0.36[ − 0.57, − 0.15]	0.0009	NP	NP	NP
	Sotagliflozin	5	1787/1260	− 0.41[ − 0.46, − 0.36]	< 0.00001	0.00	0	0.48
FPG (mmol/L)	Overall	14	4266/2364	− 1.15 [ − 1.37, − 0.93]	< 0.0001	0.00	0	0.79
	Canagliflozin	1	234/117	− 0.55 [ − 1.85, 0.75]	0.41	NP	NP	NP
	Dapagliflozin*	2	904/436	− 1.30 [ − 1.81, − 0.79]	< 0.0001	0.00	0	0.65
	Empagliflozin	4	1195/482	− 1.22 [ − 1.90, − 0.53]	0.0005	0.00	0	0.86
	Ipragliflozin	2	146/69	− 1.72 [ − 4.03, 0.59]	0.14	1.46	51	0.15
	Sotagliflozin	5	1787/1260	− 1.12 [ − 1.40, − 0.83]	< 0.00001	0.01	7	0.36
Body weight (kg)	Overall	14	4275/2376	− 2.37 [ − 2.82, − 1.92]	< 0.00001	0.55	82	< 0.00001
	Canagliflozin	1	234/117	− 3.60 [ − 5.08, − 2.12]	< 0.00001	NP	NP	NP
	Dapagliflozin	3	1018/484	− 2.44 [ − 3.58, − 1.30]	< 0.0001	0.75	77	0.01
	Empagliflozin	4	1195/482	− 2.25 [ − 2.95, − 1.55]	< 0.0001	0.27	54	0.09
	Ipragliflozin	2	146/69	− 1.27 [ − 2.64, 0.09]	0.07	0.67	64	0.10
	Sotagliflozin	4	1682/1224	− 2.72 [ − 3.09, − 2.35]	< 0.00001	0.07	52	0.10
Daily total insulin dosage (IU/d)	Overall	15	4387/2404	− 5.83[ − 6.62, − 5.04]	< 0.00001	0.00	0	0.79
	Canagliflozin	1	234/117	− 5.85 [ − 12.39, 0.69]	0.08	NP	NP	NP
	Dapagliflozin	3	1025/476	− 6.47 [ − 8.42, − 3.21]	< 0.00001	0.00	0	0.97
	Empagliflozin	4	1195/482	− 6.19 [ − 7.99, − 4.38]	< 0.00001	0.02	2	0.38
	Ipragliflozin	2	146/69	− 7.24 [ − 8.84, − 5.64]	< 0.00001	0.00	0	0.79
	Sotagliflozin	5	1787/1260	− 4.90 [ − 6.09, − 3.72]	< 0.00001	0.00	0	0.98
Daily basal insulin dosage (IU/kg/d)	Overall	13	3404/1941	− 2.88[ − 3.55, − 2.21]	< 0.00001	0.47	39	0.07
	Canagliflozin	1	234/117	− 4.80 [ − 8.09, − 1.51]	0.004	NP	NP	NP
	Dapagliflozin	1	42/13	− 6.13 [ − 11.23, − 1.03]	0.02	NP	NP	NP
	Empagliflozin	4	1195/482	− 3.46 [ − 4.56, − 2.36]	< 0.00001	0.00	0	0.71
	Ipragliflozin	2	146/69	− 3.73 [ − 4.62, − 2.84]	< 0.00001	0.00	0	0.62
	Sotagliflozin	5	1787/1260	− 2.02 [ − 2.61, − 1.43]	< 0.00001	0.00	0	0.60
Daily bolus insulin dosage (IU/kg/d)	Overall	13	3404/1941	− 3.19 [-3.85, − 2.52]	< 0.00001	0.00	0	0.99
	Canagliflozin	1	234/117	− 1.70 [ − 6.90, 3.50]	0.52	NP	NP	NP
	Dapagliflozin	1	42/13	− 0.56 [ − 10.81, 9.69]	0.91	NP	NP	NP
	Empagliflozin	4	1195/482	− 3.39 [ − 4.65, − 2.12]	< 0.0001	0.00	0	0.83
	Ipragliflozin	2	146/69	− 3.50 [ − 4.86, − 2.14]	< 0.00001	0.00	0	0.99
	Sotagliflozin	5	1787/1260	− 2.98 [ − 3.96, − 2.00]	< 0.00001	0.00	0	0.77
Seated systolic blood pressure (mmHg)	Overall	11	2345/1017	− 3.15 [ − 4.19, − 2.11]	< 0.00001	0.00	0	0.96
	Dapagliflozin*	2	511/226	− 3.22 [ − 5.25, − 1.20]	< 0.00001	0.00	0	0.57
	Empagliflozin	4	1195/482	− 2.79 [ − 4.85, − 0.72]	0.002	0.00	0	0.62
	Ipragliflozin	1	31/10	− 3.85 [ − 11.31, 3.61]	0.31	NP	NP	NP
	Sotagliflozin	3	608/299	− 3.28 [ − 4.81, − 1.74]	< 0.0001	0.00	0	0.68
Seated diastolic blood pressure (mmHg)	Overall	10	3165/1778	− 1.59 [ − 1.98, − 1.20]	< 0.00001	0.01	12	0.33
	Dapagliflozin*	1	518/260	− 1.05 [ − 2.46, 0.36]	0.15	NP	NP	NP
	Empagliflozin	4	1309/506	− 1.16 [ − 2.33, 0.01]	0.05	0.00	0	0.75
	Ipragliflozin	1	31/10	− 3.85 [ − 11.31, 3.61]	0.31	NP	NP	NP
	Sotagliflozin	5	1307/1002	− 1.52 [ − 2.24, − 0.80]	< 0.0001	0.23	50	0.11

*FPG*, fasting plasma glucose; HbA1c, hemoglobin A1c; SGLT2i, sodium–glucose cotransporter inhibitor; T1DM, type 1 diabetes; NP not reported. Follow-up at 24–26 weeks as a priority. * DEPICT-1 Trial at 52 weeks

### Subgroup analysis by SGLT2 inhibitors

We also observed the efficacy of different SGLT2is, including canagliflozin, dapagliflozin, empagliflozin, ipragliflozin, and sotagliflozin (Table 1). Insulin treatment plus dapagliflozin, empagliflozin, or sotagliflozin markedly decreased %HbA1c, FPG, body weight, daily insulin dose (total, basal, and bolus), and seated systolic blood pressure. Canagliflozin which is a drug significantly lowered %HbA1c, body weight, and daily basal insulin dose, but demonstrated no significant effect on body weight or daily insulin dose (total, bolus). Only two RCTs on ipragliflozin were conducted in Japan, with a small sample size, and ipragliflozin was shown to abate %HbA1c and daily insulin dose (total, basal, and bolus). Interestingly, only sotagliflozin significantly decreased SBP (by − 1.52 mmHg [ − 2.24, − 0.80], *P* < 0.001) and empagliflozin slightly changed SBP (by − 1.14 [ − 2.36, 0.07], *P* = 0.06).

### Subgroup analysis by the duration of follow-up

According to the duration of follow-up, the subgroup analysis from the consolidation of ranges included four periods: 1–4, 12–18, 24–26, and 52 weeks (Table 2). Two studies had follow-ups of only 12–18 weeks. We found that different follow-up periods in different outcome measures had a great impact on the results. The combination of SGLT2 inhibitors and insulin treatment reduced %HbA1c and body weight at all follow-up durations (*P* < 0.05). An SGLT2 inhibitor plus insulin treatment reduced FPG and total, basal, and bolus insulin doses at 1–4, 24–26, and 52 weeks (*P* < 0.05) not at 12–16 weeks. Both SBP and DBP markedly decreased at 24–26 weeks (*P* < 0.001). At 52 weeks, the combination treatment significantly decreased seated systolic blood pressure ( − 3.29[ − 4.37, − 2.21], *P* < 0.001) but only slightly decreased diastolic blood pressure ( − 1.73[ − 2.14, − 1.32], *P* = 0.06).

**Table 2:** Subgroup analysis on efficacy changes by follow-up comparing SGLT2i with placebo in T1DM patients with insulin treatment

Comparison		No. of studies	Participants (SGLT2i/placebo)	Overall effect		Heterogeneity		
Outcome	Subgroup			Pooled results (95% CI)	*P*	Tau^2^	I^2^, %	*P*
GHbA1c (%)	1–4 weeks	3	109/47	− 0.28[ − 0.50, − 0.06]	< 0.00001	0.00	0	0.37
	12–18 weeks	2	339/153	− 0.37[ − 0.51, − 0.24]	0.01	0.00	0	0.91
	24-26 weeks	8	3841/2186	− 0.41[ − 0.45, − 0.37]	< 0.00001	0.00	0	0.77
	52 weeks	5	2214/1105	− 0.29[ − 0.35, − 0.23]	< 0.00001	0.00	0	0.59
FPG (mmol/L)	1–4 weeks	5	182/70	− 2.21[ − 3.68, − 0.74]	0.003	0.00	0	0.87
	12–18 weeks	2	339/153	− 0.28[ − 0.50, − 0.06]	0.01	0.00	0	0.91
	24-26 weeks	6	2883/1718	− 1.12[ − 1.36, − 0.87]	< 0.00001	0.00	0	0.55
	52 weeks	5	2214/1105	− 1.07[ − 1.47, − 0.67]	< 0.00001	0.07	35	0.19
Body weight (kg)	1–4 weeks	5	182/70	− 1.33[ − 1.92, − 0.75]	< 0.00001	0.19	44	0.13
	12–18 weeks	1	234/117	− 3.60[ − 5.08, − 2.12]	< 0.00001	NP	NP	NP
	24-26 weeks	8	3824/2189	− 2.85[ − 3.06, − 2.63]	< 0.00001	0.01	12	0.34
	52 weeks	5	2238/1115	− 3.40[ − 3.98, − 2.81]	< 0.00001	0.22	57	0.05
Daily total insulin dose (IU/d)	1–4 weeks	5	182/70	− 5.40[ − 9.08, − 1.72]	0.004	0.00	0	0.83
	12–18 weeks	2	339/153	− 4.61[ − 9.88, 0.65]	0.09	0.00	0	0.53
	24-26 weeks	8	3641/2181	− 5.85[ − 6.76, − 4.95]	< 0.00001	0.22	13	0.33
	52 weeks	5	2258/1112	− 5.62 [ − 7.42, − 3.82]	< 0.00001	1.97	48	0.10
Daily basal insulin dose (IU/d)	1–4 weeks	5	182/70	− 2.96[ − 4.52, − 1.41]	0.0002	0.00	0	0.69
	12–18 weeks	2	339/153	− 4.08[ − 7.00, − 1.15]	0.006	3.44	0	0.34
	24-26 weeks	6	2883/1718	− 2.81[ − 3.68, − 1.93]	< 0.00001	0.77	68	0.008
	52 weeks	3	1310/669	− 2.96[ − 4.21, − 1.71]	< 0.00001	0.68	58	0.09
Daily bolus insulin dose (IU/d)								
	1–4 weeks	5	182/70	− 3.57[ − 6.58, − 0.57]	0.02	0.00	0	0.92
	12–18 weeks	2	339/153	− 1.42[ − 6.20, − 3.36]	0.56	0.00	0	0.29
	24-26 weeks	6	2883/1718	− 3.17[ − 3.86, − 2.48]	< 0.00001	0.00	0	0.89
	52 weeks	3	1310/669	− 2.45[ − 4.15, − 0.75]	0.005	1.15	51	0.15
Seated systolic blood pressure (mmHg)	1–4 weeks	3	150/43	− 2.58[ − 5.15, − 0.01]	0.05	0.00	0	0.50
	24-26 weeks	5	2768/1659	− 2.95[ − 3.90, − 2.00]	< 0.00001	0.00	0	0.40
	52 weeks	4	1764/882	− 3.29[ − 4.37, − 2.21]	< 0.00001	0.00	0	0.93
Seated diastolic blood pressure (mmHg)	1–4 weeks	2	93/30	− 0.60[ − 2.95, 1.76]	0.62	0.00	0	0.54
	24-26 weeks	5	2768/1659	− 1.44[ − 2.00, − 0.89]	< 0.00001	0.17	52	0.10
	52 weeks	4	1004/498	− 1.73[ − 2.14, − 1.32]	0.06	0.00	0	0.70

*FPG*, fasting plasma glucose; HbA1c, hemoglobin A1c; SGLT2i, sodium–glucose cotransporter inhibitor; T1DM, type 1 diabetes; NP not reported

### Subgroup analysis of placebo-subtracted differences between 24–26 and 52 weeks

**Fig. 2:**
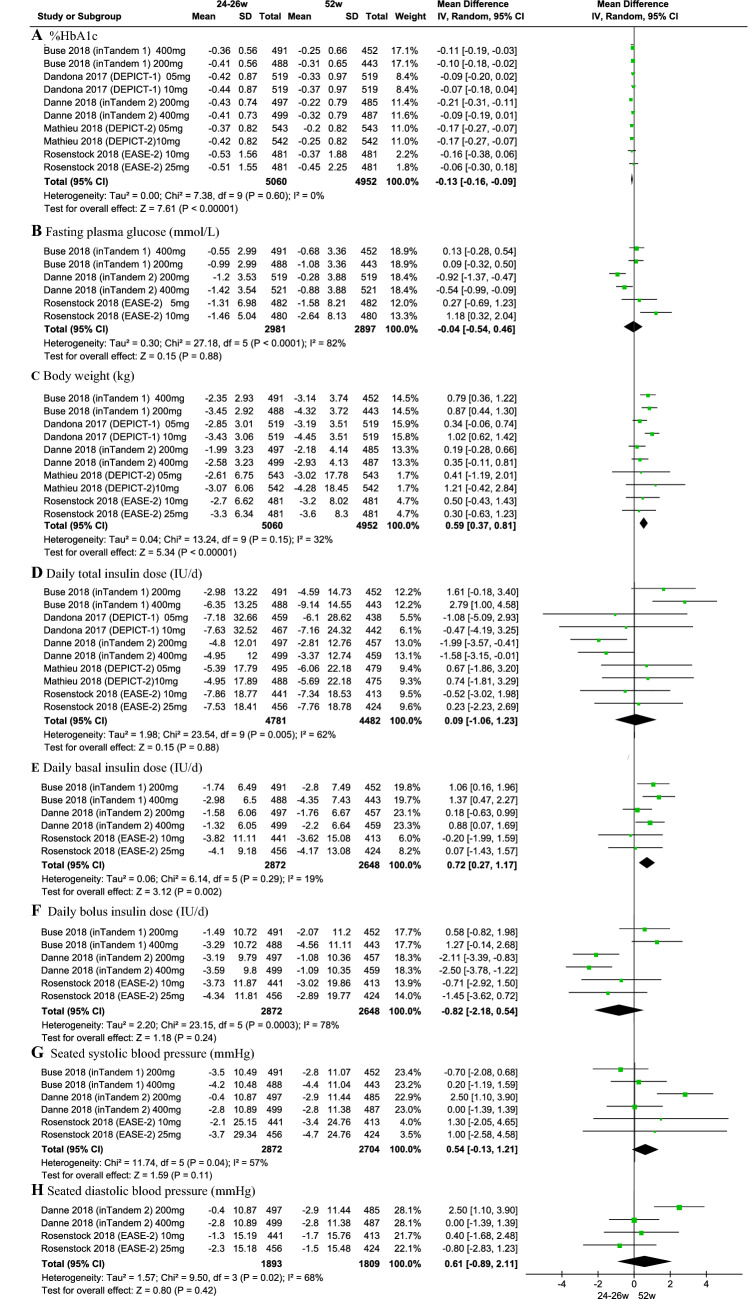
Effects of SGLT2 inhibitors on placebo-subtracted differences from baseline between 24–26 and 52 weeks

To further explore the long-term effects of SGLT2is on T1DM, we compared the placebo-subtracted difference between 24–26 and 52 weeks among the five trials [23, 25, 29, 31, 32, 34]. In the five trials, two trials were on sotagliflozin, two trials on dapagliflozin, and one trial on empagliflozin. The placebo-subtracted difference in body weight at 52 weeks was further reduced by 0.60 kg ([0.37, 0.82]; *P* < 0.001) and daily basal insulin dose (0.72 [0.27, 1.17]; *P* = 0.002), while the placebo-subtracted difference in %HbA1c increased by ( − 0.11 [ − 0.14, − 0.07]; *P* < 0.001) (Fig. 2).

### Subgroup analysis of changes in SGLT2i or placebo groups between 24–26 and 52 weeks

Similarly, in the SGLT2i groups and relative to the baseline level, changes in %HbA1c at 24–26 weeks were larger than those at 52 weeks ( − 0.18 [ − 0.22, − 0.13]; *P* < 0.001) (Fig. S3). Compared with 24–26 weeks, body weight at 52 weeks was also slightly reduced by 0.26 kg ([0.01, 0.50]; *P* = 0.04)). In addition, we also compared the changes in insulin monotherapy groups between 24–26 and 52 weeks (Fig. S4). Surprisingly, we found that body weight and seated systemic blood pressure at 52 weeks increased 0.35 kg ([ − 0.69, − 0.01]; *P* = 0.04) and − 1.83 mmHg [ − 3.14, − 0.51]; *P* = 0.007), respectively, and %HbA1c also slightly increased − 0.07 ([ − 0.14, − 0.00]; *P* = 0.05). These results demonstrated that there was weight gain, increased blood pressure, and poor glycemic control in studies of long-term insulin monotherapy in T1DM.

### Safety of SGLT-2 inhibitor intervention

A summary of the overall safety and selected AEs is shown in Table 3. Regarding the comparisons of the plus treatment between the SGLT2i groups and placebo groups, the differences in the incidence of AEs were significant, with an RR of 1.20 ([1.05, 1.38], *P* = 0.008) at 24–26 weeks and 1.43 ([1.21, 1.69], *P* < 0.001) at 52 weeks relative to the placebo group. The rates of serious AEs were also higher for the combination treatment group, with an RR of 6.37 ([1.24, 32.62], *P* = 0.03) at 12–18 weeks, 1.54 ([1.14, 2.08], *P* = 0.005) at 24–26 weeks, and 1.40 ([1.12, 1.77], *P* = 0.004) at 52 weeks relative to the placebo.

**Table 3:** Safety of SGLT2i compared with placebo in T1DM patients with insulin treatment by follow-up

Comparison		No. of studies	Events/participants		Overall effect		Heterogeneity		
Outcome	Subgroup		SGLT2i + Insulin	Placebo + Insulin	Pooled results (95% CI)	*P*	Tau^2^	*I*^2^, %	*P*
Adverse events (AEs) (N)	1–4 weeks	5	156/198	60/71	0.81[0.36, 1.82]	0.61	2.85	0	0.58
	12–18 weeks	2	183/339	82/153	1.08[0.73, 1.58]	0.71	3.19	69	0.07
	24-26 weeks	5	1801/2662	929/1536	1.20[1.05, 1.38]	0.008	2.20	0	0.70
	52 weeks	5	2123/2595	991/1301	1.43[1.21, 1.69]	< 0.00001	13.08	69	0.01
Serious adverse events (SAEs) (N)	1–4 weeks	5	3/198	3/71	0.49[0.15, 1.64]	0.25	5.40	44	0.14
	12–18 weeks	2	20/339	1/153	6.37[1.24, 32.62]	0.03	2.99	67	0.08
	24-26 weeks	5	166/2662	61/1536	1.54[1.14, 2.08]	0.005	8.03	50	0.09
	52 weeks	5	300/2595	111/1301	1.40[1.12, 1.77]	0.004	0.60	0	0.96
Urinary tract infection (UTI)	1–4 weeks	3	3/150	0/43	1.14[0.12, 10.64]	0.91	0.00	0	0.95
	12–18 weeks	2	12/339	4/153	1.40[0.45, 4.32]	0.56	3.82	74	0.05
	24-26 weeks	5	124/2662	71/1535	0.95[0.70, 1.28]	0.72	4.32	7	0.36
	52 weeks	4	171/2054	82/1029	1.05[0.80, 1.38]	0.73	0.28	0	0.96
Genital tract infection (GTI)	1–4 weeks	4	3/150	0/43	1.15[0.12, 10.66]	0.90	0.13	0	0.72
	12–18 weeks	2	12/339	4/153	2.28[0.70, 7.36]	0.17	0.00	0	0.95
	24-26 weeks	4	166/1928	27/1294	4.14[2.72, 6.29]	< 0.00001	1.29	0	0.73
	52 weeks	4	327/2054	44/1270	4.37[3.15, 6.06]	< 0.00001	1.22	0	0.75
Documented hypoglycemia, patient (N)	1–4 weeks	5	155/167	48/54	1.72[0.68, 4.38]	0.25	6.42	53	0.09
	12–18 weeks	2	330/339	148/153	1.36[0.44, 4.21]	0.60	1.74	43	0.19
	24-26 weeks	6	2151/2662	1315/1535	1.02[0.84, 1.22]	0.88	2.88	0	0.58
	52 weeks	4	1322/2054	656/1027	1.11[0.88, 1.40]	0.38	13.71	85	0.001
Severe hypoglycemia, patient (N)	1–4 weeks	5	1/183	0/43	0.98[0.04, 25.40]	0.99	NP	NP	NP
	12–18 weeks	2	14/339	2/153	2.76[0.71, 10.76]	0.56	0.00	0	0.94
	24-26 weeks	5	111/2662	62/1536	0.99[0.72, 1.37]	0.95	0.00	0	0.77
	52 weeks	4	107/2054	61/1027	0.87[0.62, 1.21]	0.40	2.84	0	0.42
Ketone-related SAE Diabetic ketoacidosis (DKA)	1–4 weeks	2	6/69	0/22	2.44[0.28, 21.09]	0.42	0.00	0	0.82
	12–18 weeks	2	19/339	0/153	6.19[0.53, 72.47]	0.15	0.91	29	0.24
	24-26 weeks	5	127/2662	29/1536	2.47[0.79, 7.72]	0.12	1.23	80	0.0004
	52 weeks	4	174/2070	25/1033	0.47 [0.62, 1.21]	0.003	0.80	74	0.0002
	1–4 weeks	2	2/2054	0/1029	6.03[0.27, 135.99]	0.26	NP	NP	NP
	12–18 weeks	2	13/339	0/153	4.14[0.30, 56.40]	0.29	1.18	33	0.22
	24-26 weeks	5	47/2662	9/1536	2.51[1.23, 5.15]	0.01	0.00	0	0.01
	52 weeks	5	97/2547	11/1270	3.94[1.81, 8.58]	0.0006	0.19	24	0.26

*FPG*, fasting plasma glucose; HbA1c, hemoglobin A1c; SGLT2i, sodium–glucose cotransporter inhibitor; T1DM, type 1 diabetes; *NP* not reported

Several specific AEs occurred more frequently than others, such as ketone-related AEs, DKA, hypoglycemia, urinary tract infections, and genital mycotic infection. We found no significant difference between the combination group and the monotherapy group for hypoglycemia, severe hypoglycemia, or UTIs (*P* > 0.05). However, compared with placebo, SGLT2i treatment was associated with a significantly higher rate of GTIs at 24–26 weeks (4.14[2.72, 6.29], *P* < 0.001) and 52 weeks (4.37 [3.15, 6.06], *P* < 0.001)] in patients with T1DM receiving insulin therapy (Table 3). Interestingly, at 52 weeks, we noted significantly increased risks of ketone-related SAEs and DKA (0.47[0.62, 1.21], 3.94[1.81, 8.58], respectively) in the SGLT2i plus insulin groups compared with those in the insulin monotherapy group, but we noted no effects at other follow-up time points.

## Discussion

### Main findings

In our study, initial combination therapy with a SGLT2i and insulin was more efficacious in terms of glycemic control, body weight, and seated blood pressure control than treatment with insulin alone in T1DM. However, the subgroup analysis by the length of follow-up also showed that SGLT2is as add-on therapy to insulin did provide insulin-independent glucose lowering after 6 months, but the effects might weaken. With the extension of the follow-up, especially at 52 weeks, the frequency of genital infections and DKA significantly increased in T1DM patients treated with SGLT2is as an adjunct to insulin. The above findings warrant careful consideration of long-term benefits and potential undesirable effects of these SGLT2is as add-on treatment to insulin.

In our study, subgroup analysis showed that short-term (1–4 weeks) to long-term (52 weeks) SGLT2is plus insulin resulted in a larger reduction in HbA1c and FPG levels compared with placebo in T1DM patients. In a meta-analysis, SGLT2is ameliorated glycemic efficacy outcomes accompanied by a lower insulin dose requirement without increasing the risk of hypoglycemia [40]. Importantly, SGLT2is improving glycemic control by increasing time in range on average while reducing glycemic variability [41, 42]. SGLT2is are particularly attractive for add-on therapy to insulin in T1DM because they are oral agents that decrease the reabsorption of glucose in the kidney and increase its excretion via the urine, a mechanism that is not dependent of islet cell functionality [14–16, 43, 44]. In addition, sotagliflozin, a dual SGLT1 and SGLT2 inhibitor, can delay and blunt intestinal glucose absorption after meals, resulting in lower PPG and insulin levels [45].

Consistent with previous studies, our study confirmed that weight gain may be the side effect of long-term insulin monotherapy in T1DM, which is the main reason that intensive insulin therapy fails to improve the microvascular and macrovascular complications of diabetes [1, 2, 46–48]. So noninsulin pharmacological therapies as an add-on treatment to insulin have received a surge of interest in T1DM patients. We found that SGLT2is not only offset the weight gain induced by insulin treatment but also reduced the blood pressure. Osmotic diuresis and natriuresis are the reasons for weight loss, which is also the reason for the improvement in blood pressure [17, 49]. The use of a SGLT2i as an add-on therapy to insulin may be a preferred option for patients with T1DM.

In addition to insulin side effects, another disadvantage of insulin replacement treatment for T1DM patients is the lack of a longer-term glycemic benefit [50]. To explore the longer-term glycemic effects, we first compared them with four trials to simultaneously investigate the efficacy at 24–26 and 52 weeks in the same population [23, 25, 29, 31, 32, 34]. Notably, the placebo-subtracted difference and changes in %HbA1c in the SGLT2i group at 24–26 weeks were larger than those at 52 weeks (Fig. 2). Furthermore, we also found that decreases from 24–26 to 52 weeks were dose-independent for the different SGLT2is (including dapagliflozin, sotagliflozin, and empagliflozin) [23, 25, 29, 31, 32, 34]. This phenomenon shows that the addition of an SGLT2i to insulin did provide insulin-independent glucose lowering after 6 months, but the effects weaken, which seems to be a contradiction because an SGLT2i as an add-on to metformin treatment gradually reduced %HbA1c from 24 to 104 weeks in T2DM patients [51, 52]. In this regard, the 52-week study period may have been too brief to show longer beneficial effects of SGLT2is on glycemic control. A possible mechanism for the contradiction is lower renal threshold for glucose reabsorption in T1DM patients [53, 54]. The renal threshold for glucose reabsorption in T1DM patients with T1DM was near the normal range and significantly lower than that in T2DM patients [53].

In our meta-analysis, genital tract infections (GTIs) occurred more often in SGLT2i plus insulin therapy than in insulin monotherapy, and there was no difference in the occurrence of urinary tract infections (UTIs). Subgroup analysis on treatment duration showed that the effects of SGLT inhibition plus insulin on safety outcomes were duration-dependent, although there were slight effects at 1–4 and 12–18 weeks. This is consistent with the previous literature that SGLT2is increase the risk of GTIs [55, 56], but we first reported that a longer duration might confer a higher risk of GTI events in T1DM.

Recently, the FDA warned that SGLT2is can produce too many ketoacids in some diabetes patients [57]. Our meta-analysis also demonstrated that the use of SGLT2i as an add-on therapy was associated with long-term risks in the incidence of ketone-related SAEs and DKA in T1DM patients receiving insulin therapy.

Therefore, to sufficiently comprehend the treatment benefits and risks of SGLT2is over a long period of follow-up, future RCTs should be more effective.

### Comparison with other studies

Five previous meta-analyses reported that an SGLT2i as an add-on therapy to insulin is effective in improving glycemic and blood pressure control and decreasing body weight and total daily insulin dose in patients with T1DM [35, 40, 58–60]. Four of these meta-analyses researched studies evaluating the use of SGLT2is in patients with T1DM before 2018 [40, 58–60]. In addition, two meta-analyses confirmed that dual SGLT 1/2 inhibitor sotagliflozin adjuvant therapy improves glycemic and nonglycemic outcomes and reduces the rate of hypoglycemia and severe hypoglycemia [37, 61].

In contrast, two early meta-analyses reported that, compared with a control treatment, SGLT2 inhibitors did not increase the risk of adverse events [58, 59]. A third study reported that only the risk of DKA should be carefully monitored in SGLT2 inhibitors as adjunctive therapy [50, 60]. The latest four studies confirmed that add-on SGLT-2i therapy increased diabetic ketoacidosis and genital tract infections [35, 37, 40, 61].

In our study, we identified eligible RCTs from inception through May 2020, including 15 trials of 7,109 patients. Our results regarding the efficacy and safety of SGLT2i as an add-on therapy were generally consistent with previous findings. However, we assessed the safety and efficacy of SGLT2is (including canagliflozin, dapagliflozin, empagliflozin, sotagliflozin, and ipragliflozin) at different follow-up periods through subgroup analysis. In addition to the data on efficacy in long-term treatment, we also analyzed events suggestive of effective outcomes in the same four RCTs regardless of biases.

### Limitations

Our study also has some limitations. Although the majority of trials used the same classification system for efficacy and safety, some other trials may have overreported adverse events using symptoms alone. Second, a wide variation in the duration of follow-up of the included studies was noted, from 1 to 52 weeks. Third, for the outcomes of efficacy and safety, trials of dapagliflozin, canagliflozin, empagliflozin, sotagliflozin, and ipragliflozin accounted for the majority of the evidence. Long-term treatment merely focused on empagliflozin, sotagliflozin, and dapagliflozin, and the follow-up of only four studies reached 52 weeks. RCTs on canagliflozin or ipragliflozin were few and had a small sample sizes [50].

## Conclusion

In summary, SGLT2is as adjunctive therapy improved glycemic control and body weight and decreased the required dose of insulin without increasing the risk of hypoglycemia. However, the subgroup analysis by the length of follow-up also showed that SGLT2is as add-on therapy to insulin did provide insulin-independent glucose lowering after 6 months, but the effects might weaken.

## Supplementary information

Below is the link to the electronic supplementary material.

Supplementary file1 (EPS 518 kb)

Below is the link to the electronic supplementary material.

Supplementary file1 (EPS 201 kb)

Below is the link to the electronic supplementary material.

Supplementary file1 (EPS 6985 kb)

Below is the link to the electronic supplementary material.

Supplementary file1 (EPS 4792 kb)

Below is the link to the electronic supplementary material.

Supplementary file1 (DOCX 68 kb)

Below is the link to the electronic supplementary material.

Supplementary file1 (DOCX 54 kb)
