# Supplementary material for: Sodium–glucose cotransporter 2 inhibitors as an add-on therapy to insulin for type 1 diabetes mellitus: Meta-analysis of randomized controlled trials
Source: Acta Diabetol. 2021 Mar 2;58(7):869–80. doi: 10.1007/s00592-021-01686-x (PMC8187227; doi:10.1007/s00592-021-01686-x)
Supplement: Supplementary file 5 — Supplementary file1 (DOCX 68 kb) [file 592_2021_1686_MOESM5_ESM.docx]

Table S1 Baseline characteristics of all included studies

| Study | Participants and baseline data | Intervention/outcomes |
| --- | --- | --- |
| Canagliflozin (Cana): |  |  |
| Henry 2015 C [1]  Setting: double-blind, parallel-group, multi-center, phase 2 study RCT  Cana 100 mg, Cana 300 mg vs. placebo  Follow-up: 18weeks  NCT02139943 | N: 351  Participants: patients with type 1 diabetes inadequately controlled with multiple daily injections of insulin treatment  Age (years):  Cana 100 mg 42.0, SD 11.6; Cana 300 mg 42.8, SD 11.0 or PBO 42.0, SD 11.9  HbA1c (%):  Cana 100 mg 7.9, SD 0.5; Cana 300 mg 8.0, SD 0.5 or PBO 7.9, SD 0.6  BMI (kg/m2):  Cana 100 mg 28.0, SD 3.9; Cana 300 mg 28.1, SD 3.9 or PBO 28.0, SD 3.6  Daily insulin dose, IU/kg:  Cana 100 mg 0.70; Cana 300 mg 0.62 or PBO 0.63 | Interventions  1. Cana 100 mg + Insulin (n=117)  2. Cana 300 mg + Insulin (n=117)  Comparator  Placebo + Insulin (n=117)  Outcomes  Primary outcomes: change from baseline in HbA1c and weight at 18 weeks  Other outcomes: FPG, systolic blood pressure (SBP), diastolic blood pressure (DBP) and eGFR, total, basal and bolus insulin doses at 18 weeks, and safety outcomes |
| Dapagliflozin (Dapa) |  |  |
| Henry 2015 D [2]  Design: randomized, double-blind, three-arm, placebo-controlled, parallel-group study RCT  Dapa 5 mg, Dapa 10 mg vs. placebo  Follow-up: 2 weeks  NCT01498185 | N: 70  Participants: patients with type 1 diabetes inadequately controlled with multiple daily injections of insulin treatment  Age (years):  Dapa 1 mg 33.7, SD 9.1; Dapa 2.5 mg 35.7, SD 13.9, Dapa 5 mg 34.8, SD 14.0; Dapa 10 mg 37.5, SD 15.2 or PBO 34.5, SD 12.2  HbA1c (%):  Dapa 1 mg 8.21, SD 0.68; Dapa 2.5 mg 8.45, SD 0.86, Dapa 5 mg 8.50, SD 0.78; Dapa 10 mg 8.39, SD 0.82 or PBO 8.75, SD 0.92  BMI (kg/m2):  Dapa 1 mg 25.1, SD 3.8; Dapa 2.5 mg 24.8, SD 2.7, Dapa 5 mg 23.4, SD 2.7; Dapa 10 mg 25.8, SD 4.8 or PBO 25.3, SD 3.0  Daily insulin dose, IU/kg:  Dapa 5 mg 0.76, SD 0.52; Dapa 10 mg 0.71, SD 0.26 or PBO 0.74, SD 0.25 | Interventions  1. Dapa 1 mg + Insulin (n=13)  2. Dapa 2.5 mg + Insulin (n=15)  3. Dapa 5 mg + Insulin (n=14)  4. Dapa 10 mg + Insulin (n=15)  Comparator  Placebo + Insulin (n=13)  Outcomes  Primary outcomes:  Mean change from Baseline in 7-point glucose monitoring (7-PGM) at day 7  Other outcomes: change in FPG, 24h UGE, body weight, total, basal and bolus insulin doses |
| Dandona 2017 (DEPICT-1) [3, 4]  Setting: multicentre study (n=143) in 17 countries  Design: randomized, double-blind, three-arm, placebo-controlled study RCT  Dapa 5 mg, Dapa 10 mg vs. placebo  Follow-up: 24weeks (2017) and 52 weeks (2018)  NCT02268214 | N: 833  Participants: patients with type 1 diabetes inadequately controlled with multiple daily injections of insulin treatment  Age (years):  Dapa 5 mg 41.9, SD 14.1; Dapa 10 mg 42.7, SD 14.1 or PBO 42.7, SD 13.6  HbA1c (%):  Dapa 5 mg 8.53, SD 0.71; Dapa 10 mg 8.52, SD 0.64 or PBO 8.53, SD 0.67  BMI (kg/m2):  Dapa 5 mg 28.3, SD 5.8; Dapa 10 mg 28.1, SD 5.1 or PBO 28.6, SD 5.2  Daily insulin dose, IU/kg:  Dapa 5 mg 0.76, SD 0.52; Dapa 10 mg 0.71, SD 0.26 or PBO 0.74, SD 0.25 | Interventions  1. Dapa 5 mg + Insulin (n=259)  2. Dapa 10 mg + Insulin (n=259)  Comparator  Placebo + Insulin (n=260)  Outcomes  Primary outcomes: change from baseline in HbA1c at 24 weeks  Other outcomes: change in FPG, 24h UGE, body weight, systolic blood pressure (SBP), diastolic blood pressure (DBP), total, basal and bolus insulin doses, and safety outcomes |
| Mathieu 2018 (DEPICT-2) [5, 6]  Setting: multicentre study (n=148) in 13 countries  Design: randomized, double-blind, three-arm, placebo-controlled study RCT  Dapa 5 mg, Dapa 10 mg vs. placebo  Follow-up: 24weeks (2018) and 52 weeks (2020)  NCT02460978 | N: 813  Participants: patients with type 1 diabetes inadequately controlled with multiple daily injections of insulin treatment  Age (years):  Dapa 5 mg 42.7, SD 13.35; Dapa 10 mg 42.4, SD 12.80 or PBO 43.0, SD 13.73  HbA1c (%):  Dapa 5 mg 8.45, SD 0.69; Dapa 10 mg 8.39, SD 0.67 or PBO 8.40, SD 0.63  BMI (kg/m2):  Dapa 5 mg 27.27, SD 5.13; Dapa 10 mg 27.80, SD 5.53 or PBO 27.62, SD 5.41  Daily insulin dose, IU/kg:  Dapa 5 mg 0.73, SD 0.26; Dapa 10 mg 0.73, SD 0.27 or PBO 0.71, SD 0.24 | Interventions  1. Dapa 5 mg + Insulin (n=271)  2. Dapa 10 mg + Insulin (n=270)  Comparator  Placebo + Insulin (n=272)  Outcomes  Primary outcomes: change from baseline in HbA1c at 24 weeks  Other outcomes: change in FPG, 24h UGE, body weight, systolic blood pressure (SBP), diastolic blood pressure (DBP), total, basal and bolus insulin doses, and safety outcomes |
| Empagliflozin (Empa) |  |  |
| Pieber 2015 (EASE-1) [7]  Setting: one centre in Germany and one centre in Austria  Design: randomized, double-blind, placebo-controlled, parallel-group RCT  Empa 2.5 mg, Empa 10 mg, Empa 25 mg vs. placebo  Follow-up: 28 days  NCT01969747 | N: 75  Participants: patients with type 1 diabetes inadequately controlled with multiple daily injections of insulin treatment  Age (years):  Empa 2.5 mg 41.9, SD 12.4; Empa 10 mg 39.6, SD 11.6; Empa 25 mg 41.9, SD 9.7 or PBO 40.5, SD 10.6  HbA1c (%):  Empa 2.5 mg 8.35, SD 0.75; Empa 10 mg 8.28, SD 0.79; Empa 25 mg 8.15, SD 0.54 or PBO 8.18, SD 0.67  BMI (kg/m2):  Empa 2.5 mg 24.7, SD 3.6; Empa 10 mg 27.4, SD 3.5; Empa 25 mg 25.4, SD 3.5 or PBO 25.4, SD 3.7  Daily insulin dose, IU/kg:  Empa 2.5 mg 0.65, SD 0.17; Empa 10 mg 0.71, SD 0.18; Empa 25 mg 0.65, SD 0.23 or PBO 0.66, SD 0.23 | Interventions  1.Empa 2.5 mg + Insulin (n=19)  2.Empa 10 mg + Insulin (n=19)  3.Empa 25 mg + Insulin (n=18)  Comparator  Placebo + Insulin (n=19)  Outcomes  Primary outcomes: change in urinary glucose excretion (UGE) from baseline to 7 days  Other outcomes: change in UGE from baseline to 4 weeks change in HbA1c percentage, FPG, mean daily glucose (MDG), body weight systolic blood pressure (SBP), diastolic blood pressure (DBP) and eGFR, total, basal and bolus insulin doses at 28 days, and safety outcomes |
| Akira 2018 [8]  Setting: multicentre (n=4) in Japanese  Design: double-blind, randomized, placebo-controlled trial RCT  Empa 2.5 mg, Empa 10 mg, Empa 25 mg vs. placebo  Follow-up: 7 days; 4 weeks  NCT02702011 | N: 48  Participants: patients with type 1 diabetes inadequately controlled with multiple daily injections of insulin treatment  Age (years):  Empa 2.5 mg 44.2, SD 12.6; Empa 10 mg 44.5, SD 11.8; Empa 25 mg 46.6, SD 10.8 or PBO 43.9, SD 11.7  HbA1c (%):  Empa 2.5 mg 8.02, SD 0.36; Empa 10 mg 8.12, SD 0.37; Empa 25 mg 7.89, SD 0.91 or PBO 8.23, SD 0.47  BMI (kg/m2):  Empa 2.5 mg 24.4, SD 3.93; Empa 10 mg 22.68, SD 3.27; Empa 25 mg 22.6, SD 2.7 or PBO 23.7, SD 2.6  Daily insulin dose, IU/kg:  Empa 2.5 mg 0.73, SD 0.17; Empa 10 mg 0.73, SD 0.23; Empa 25 mg 0.66, SD 0.15 or PBO 0.71, SD 0.22 | Interventions   1. Empa 2.5 mg + Insulin (n=13) 2. Empa 10 mg + Insulin (n=12) 3. Empa 25 mg + Insulin (n=12)   Comparator  Placebo + Insulin (n=11)  Outcomes  Primary outcomes: change in urinary glucose excretion (UGE) from baseline to 24 weeks  Other outcomes: change in FPG, acetoacetic acid, total ketone, bodies and free fatty acids (FFA), systolic blood pressure (SBP), diastolic blood pressure (DBP) and eGFR, total, basal and bolus insulin doses |
| Rosenstock 2018 (EASE-2) [9]  Setting: international (17 countries), multi-center (n=131)  Design: randomized, double-blind, placebo- controlled, parallel-group RCT Empa 10 mg, Empa 25 mg vs. placebo  Follow-up: 26 and 52 weeks  NCT0 2414958 | N: 730  Participants: patients with type 1 diabetes inadequately controlled with multiple daily injections or pump of insulin treatment  Age (years):  Empa 10 mg 45.7, SD 12.5; Empa 25 mg 45.3, SD 13.9 or PBO 44.5, SD 13.5  HbA1c (%):  Empa 10 mg 8.1, SD 0.6; Empa 25 mg 8.06, SD 0.53 or PBO 8.13, SD 0.57  BMI (kg/m2):  Empa 10 mg 29.5, SD 5.5; Empa 25 mg 29.5, SD 6.0 or PBO 28.5, SD 5.3  Daily insulin dose, IU/kg:  Empa 10 mg 0.70, SD 0.24; Empa 25 mg 0.74, SD 0.26 or PBO 0.70, SD 0.23 | Interventions  1. Empa 10 mg + Insulin (n=243)  2. Empa 25 mg + Insulin (n=244)  Comparator  Placebo + Insulin (n=243)  Outcomes  Primary outcomes: change from baseline in HbA1c at 26 weeks  Other outcomes: percentage of time spent in target glucose range, FPG, body weight, systolic blood pressure (SBP), diastolic blood pressure (DBP) and eGFR, total, basal and bolus insulin doses at 26 and 52 weeks, and safety outcomes |
| Rosenstock 2018 (EASE-3) [9]  Setting: international (24 countries) , multi-center (n=189) ;  Design: randomized, double-blind, placebo- controlled, parallel-group RCT Empa 2.5mg, Empa 10 mg, Empa 25 mg vs. placebo  Follow-up: 26 weeks  NCT02580591 | N: 977  Participants: patients with type 1 diabetes inadequately controlled with multiple daily injections or pump of insulin treatment  Age (years):  Empa 2.5 mg 43.4, SD 14.2; Empa 10 mg 42.4, SD 13.3; Empa 25 mg 44.2, SD 13.5 or PBO 42.2, SD 13.2  HbA1c (%):  Empa 2.5 mg 8.14, SD 0.61; Empa 10 mg 8.19, SD 0.64; Empa 25 mg 8.19, SD 0.65 or PBO 8.19, SD 0.58  BMI (kg/m2):  Empa 2.5 mg 28.0, SD 4.4; Empa 10 mg 28.7, SD 5.1; Empa 25 mg 28.4, SD 5.6 or PBO 27.8, SD 5.1  Daily insulin dose, IU/kg:  Empa 2.5 mg 0.70, SD 0.24; Empa 10 mg 0.71, SD 0.24; Empa 25 mg 0.71, SD 0.24 or PBO 0.70, SD 0.24 | Interventions  Interventions  1.Empa 2.5 mg + Insulin (n=237)  2.Empa 10 mg + Insulin (n=244)  3.Empa 25 mg + Insulin (n=242)  Comparator  Placebo + Insulin (n=238)  Outcomes  Primary outcomes: change from baseline in HbA1c at 26 weeks  Other outcomes: change in HbA1c percentage, FPG, body weight systolic blood pressure (SBP), diastolic blood pressure (DBP) and eGFR, total, basal and bolus insulin doses at 26 weeks |
| Ipragliflozin (Ipra) |  |  |
| Kohei 2019A [10]  Setting: multicenter (n=12) in Japan  Design: randomized, double-blind, placebo-controlled, parallel-group study RCT  Ipra 25 mg, Ipra 50 mg, Ipra 100 mg vs. placebo  Follow-up: day 1 and 14  NCT02529449 | N: 41  Participants: patients with type 1 diabetes inadequately controlled with multiple daily injections of insulin treatment  Age (years):  Ipra 25 mg 47.2 SD 15.7, Ipra 50 mg 43.4 SD 12.3, Ipra 100 mg 41.7 SD 14.0 vs. placebo 44.8 SD 13.2;  HbA1c (%):  Ipra 25 mg 8.51 SD 0.79, Ipra 50 mg 8.45 SD 0.76, Ipra 100 mg 8.85 SD 0.72 vs. placebo 8.66 SD 0.74;  BMI (kg/m2):  Ipra 25 mg 26.56 SD 5.60, Ipra 50 mg 24.54 SD 3.77, Ipra 100 mg 24.25 SD 2.64 vs. placebo 23. 93SD 2.72;  Daily insulin dose, IU/kg:  Ipra 25 mg 0.69 SD 0.29, Ipra 50 mg 0.60 SD 0.19, Ipra 100 mg 0.79 SD 0.17 vs. placebo 0.70 SD 0.28 | Interventions  1. Ipra 25 mg + Insulin (n=9)  2. Ipra 50 mg + Insulin (n=12)  3. Ipra 100mg + Insulin (n=10)  Comparator  Placebo + Insulin (n=10)  Outcomes  Pharmacodynamics of Ipar, change in mean amplitude of glucose excursion (MAGE), FPG, total and bolus insulin doses at 4 weeks, and safety outcomes |
| Kohei 2019B [11]  Setting: multicenter (n=36) in Japan  Design: randomized, double-blind, placebo-controlled, parallel-group study RCT  Ipra 50 mg vs. placebo  Follow-up: 24w  NCT02897219 | N: 175  Participants: patients with type 1 diabetes inadequately controlled with multiple daily injections of insulin treatment  Age (years):  Ipra 50 mg 49.7 SD 13.1 vs. placebo 48.3 SD 12.8;  HbA1c (%):  Ipra 50 mg 8.68 SD 0.81 vs. placebo 8.67 SD 0.79;  BMI (kg/m2):  Ipra 50 mg 24.67 SD 2.95 vs. placebo 24.21 SD 2.82;  Daily insulin dose, IU/kg:  Ipra 50 mg 0.60 SD 0.19 vs. placebo 0.70 SD 0.28 | Interventions  Ipra 50 mg + Insulin (n=115)  Comparator  Placebo + Insulin (n=60)  Outcomes  change from baseline in HbA1c at 24 weeks  Other outcomes:  Changes in FPG, insulin doses (basal/bolus/total), each time point of the seven-point SMBG, and body weight and safety |
| Sotagliﬂozin (Sota) |  |  |
| Sands 2015 [12]  Setting: multicenter (n=3) in the U.S. and Canada  Design: randomized, double-blind, placebo-controlled, parallel-group study RCT  Sota 400 mg vs. placebo  Follow-up: 4 weeks  NCT01742208 | N: 33  Participants: patients with type 1 diabetes inadequately controlled with multiple daily injections of insulin treatment  Age (years):  Sota 400 mg 45.5 (21, 55), or PBO 34.0 (21, 57)  HbA1c (%):  Sota 400 mg 7.94, SD 0.55, or PBO 7.98, SD 0.51  BMI (kg/m2):  Sota 400 mg 27.1, SD 3.1, or PBO 26.2, SD 3.0  Daily insulin dose, IU/kg:  Sota 400 mg 0.6, or PBO 0.6 | Interventions  Sota 400 mg + Insulin (n=16)  Comparator  Placebo + Insulin (n=17)  Outcomes  Primary outcomes: change from total daily bolus insulin at 4 weeks  Other outcomes: change in mean amplitude of  glucose excursion (MAGE), FPG, total and bolus insulin doses at 4 weeks, and safety outcomes |
| Buse 2018 (inTandem 1) [13]  Setting: multicenter (n=75)in the U.S. and Canada  Design: randomized, double-blind, three-arm, placebo-controlled, parallel-group study RCT  Sota 200mg, Sota 400 mg vs. placebo  Follow-up: 24 weeks  NCT02384941 | N: 793  Participants: patients with type 1 diabetes inadequately controlled with multiple daily injections of insulin treatment  Age (years):  Sota 200 mg 46.6, SD 13.48; Sota 400 mg 46.4, SD 13.12, or PBO 45.2, SD 12.7  HbA1c (%):  Sota 200 mg 7.61, SD 0.735; Sota 400 mg 7.56, SD 0.724, or PBO 7.54, SD 0.712  BMI (kg/m2):  Sota 200 mg 29.81, SD 5.686; Sota 400 mg 29.63, SD 5.297, or PBO 29.55, SD 5.188  Daily insulin dose, IU/kg:  Sota 200 mg 0.72, SD 0.386; Sota 400 mg 0.72, SD 0.335, or PBO 0.73, SD 0.360 | Interventions  1. Sota 200 mg + Insulin (n=263)  2. Sota 400 mg + Insulin (n=262)  Comparator  Placebo + Insulin (n=268)  Outcomes  Primary outcomes: change from baseline in HbA1c at 24 weeks  Other outcomes: change in HbA1c, FPG, body weight systolic blood pressure (SBP) and diastolic blood pressure (DBP), total, basal and bolus insulin doses at 52 weeks, and safety outcomes |
| Danne 2018 (inTandem 2) 2018 [14]  Setting: multicenter (n=75) in the U.S. and Canada  Design: randomized, double-blind, three-arm, placebo-controlled, parallel-group study RCT  Sota 200mg, Sota 400 mg vs. placebo  Follow-up: 24 and 52 weeks  NCT02421510 | N: 781  Participants: patients with type 1 diabetes inadequately controlled with multiple daily injections of insulin treatment  Age (years):  Sota 200 mg 42.3, SD 13.59; Sota 400 mg 41.7, SD 13.23, or PBO 39.7, SD 13.42  HbA1c (%):  Sota 200 mg 7.74, SD 0.806; Sota 400 mg 7.71, SD 0.819, or PBO 7.79, SD 0.881  BMI (kg/m2):  Sota 200 mg 29.97, SD 5.275; Sota 400 mg 27.85, SD 4.921, or PBO 27.50, SD 5.170  Daily insulin dose, IU/kg:  Sota 200 mg 0.73, SD 0.277; Sota 400 mg 0.74, SD 0.267, or PBO 0.75, SD 0.295 | Interventions  1. Sota 200 mg + Insulin (n=261)  2. Sota 400 mg + Insulin (n=262)  Comparator  Placebo + Insulin (n=258)  Outcomes  Primary outcomes: change from baseline in HbA1c at 24 weeks  Other outcomes: change in HbA1c percentage, FPG, body weight systolic blood pressure (SBP) and diastolic blood pressure (DBP), total, basal and bolus insulin doses at 24 and 52 weeks, and safety outcomes |
| Garg 2017 (inTandem 3) [15]  Setting: multicentre study (n=133) in 19 countries  Design: randomized, double-blind, three-arm, placebo-controlled study RCT Sota 400 mg vs. placebo  Follow-up: 24 weeks  NCT02531035 | N:1402  Participants: patients with type 1 diabetes inadequately controlled with multiple daily injections of insulin treatment  Age (years):  Sota400 mg 43.3 SD 14.2, or PBO 42.4 SD 14.0;  HbA1c (%):  Sota 400 mg 8.26, SD 0.96, or PBO 8.21, SD 0.92  BMI (kg/m2):  Sota 400 mg 28.29, SD 5.13, or PBO 28.10, SD 5.18  Daily insulin dose, IU/kg:  Sota 400 mg 0.69, SD 0.28, or PBO 0.68, SD 0.29 | Interventions  Sota 400 mg + Insulin (n=699)  Comparator  Placebo + Insulin (n=703)  Outcomes  Primary outcomes:  HbA1c ≤ 7.0% at 24 weeks  Other outcomes: change in HbA1c, FPG, body weight systolic blood pressure (SBP) and diastolic blood pressure (DBP), total, basal and bolus insulin doses at 52 weeks, and safety outcomes |
| Baker 2019 (inTandem 4)[16]  Setting: multicentre study (n=17) in U.S.  Design: phase 2b, randomized, double-blind, three-arm, placebo-controlled study RCT  Sota 75, 200 or 400 mg vs. placebo  Follow-up: 12 weeks  NCT02459899 | N:141  Participants: patients with type 1 diabetes inadequately controlled with multiple daily injections of insulin treatment  Age (years):  Sota 75 mg 48.1 SD 11.3; Sota 200 mg 42.4 SD 12.0; Sota 400 mg 44.8 SD 15.4 or PBO 48.1 SD 11.3;  HbA1c (%):  Sota 75 mg 8.00 SD 0.84; Sota 200 mg 8.07 SD 0.93; Sota 400 mg 8.05 SD 0.74 or PBO 7.95 SD 0.85;  BMI (kg/m^2^):  Sota 75 mg 27.4 SD 5.0; Sota 200 mg 28.0 SD 4.7; Sota 400 mg 29.4 SD 5.8 or PBO 31.8 SD 5.8;  Daily insulin dose, IU/kg:  Sota 75 mg 0.65 SD 0.23; Sota 200 mg 0.70 SD 0.30; Sota 400 mg 0.77 SD 0.41 or PBO 0.68 SD 0.31 | Interventions  Sota 75 mg + Insulin (n=35)  Sota 200 mg + Insulin (n=35)  Sota 400 mg + Insulin (n=35)  Comparator  Placebo + Insulin (n=36)  Outcomes  Primary outcomes:  HbA1c ≤ 7.0% at 12 weeks  Other outcomes: change in HbA1c, FPG, body weight systolic blood pressure (SBP) and diastolic blood pressure (DBP), total, basal and bolus insulin doses at 12 weeks, and safety outcomes |
