# Supplementary material for: Sodium–glucose cotransporter 2 inhibitors as an add-on therapy to insulin for type 1 diabetes mellitus: Meta-analysis of randomized controlled trials
Source: Acta Diabetol. 2021 Mar 2;58(7):869–80. doi: 10.1007/s00592-021-01686-x (PMC8187227; doi:10.1007/s00592-021-01686-x)
Supplement: Supplementary file 6 — Supplementary file1 (DOCX 54 kb) [file 592_2021_1686_MOESM6_ESM.docx]

Table S2. Characteristics of the 15 studies including baseline characteristics of participants and details of the interventions.

|  | Dosage(cases) | Age (y) | Body weight (kg) | Males | BMI  (kg/m2) | Disease  length (y) | HbA1c  (%) | FPG  (mM/L) | Total insulin dose | basal insulin dose | bolus insulin dose | SBP  (mm Hg) | DBP  (mm Hg) | eGFR  mL/(min·1.73 m^2^) |
| --- | --- | --- | --- | --- | --- | --- | --- | --- | --- | --- | --- | --- | --- | --- |
| Henry 2015 C (2015) ^[^[^1^](#_ENREF_1)^]^ | Canagliflozin  100 mg: 117  300 mg: 117  Placebo: 117 | 42.27 ± 11.48 | NP | 56.10% | 28.03 ± 3.79 | 22.40 ± 11.02 | 7.93 ± 0.54 | 10.13 | 0.65 | 0.33 | 0.33 | NP | NP | 96.40 ± 15.39 |
| Henry 2015 D (2015) ^[^[^2^](#_ENREF_2)^]^ | Dapagliflozin  1 mg: 13  2.5 mg: 15  5 mg: 14  10 mg: 15  Placebo:13 | 35.31 ± 12.85 | 75.63 ± 14.72 | 57.14% | 24.88 ± 3.46 | 18.71 ± 11.32 | 8.46 ± 0.81 | 8.61 ± 3.76 | 0.71 ± 0.36 | 0.37 ± 0.24 | 0.35 ± 0.19 | NP | NP | NP |
| Dandona 2017 (DEPICT-1) (2017) ^[^[^3^](#_ENREF_3)^]^  (2018) ^[^[^4^](#_ENREF_4)^]^ | Dapagliflozin  5 mg:259  10 mg:259  Placebo:260 | 42.43 ± 13.92 | 82.37  ± 17.98 | 44.78% | 28.33 ± 5.37 | 20.27 ± 11.78 | 8.53 ± 0.67 | NP | 0.74 ± 0.37 | NP | NP | NP | NP | NP |
| Mathieu 2018 (DEPICT-2) (2018) ^[^[^5^](#_ENREF_5)^]^ (2020) ^[^[^6^](#_ENREF_6)^]^ | Dapagliflozin  5 mg:271  10 mg:270  Placebo:272 | 42.70 ± 13.29 | 79.22  ± 18.18 | 44.03% | 27.56 ± 5.36 | 19.26 ± 11.77 | 8.44 ± 0.68 | NP | 0.73 ± 0.26 | NP | NP | NP | NP | NP |
| Pieber 2015 (EASE-1) (2015) ^[^[^7^](#_ENREF_7)^]^ | Empagliflozin  2.5 mg: 19  10 mg: 19  25 mg: 18  Placebo: 19 | 40.96 ± 10.96 | 79.97 ± 14. 36 | 64.0% | 25.73 ± 3.65 | 20.3 ± 12.14 | 7.24 ± 0.69 | 9.80 ± 3.41 | 0.67 ± 0.20 | 0.33 ± 0.11 | 0.34 ± 0.15 | 123.13 ± 13.54 | 73.92 ± 8.42 | 102.37 ± 13.80 |
| Akira 2018^[^[^8^](#_ENREF_8)^]^ | Empagliflozin  2.5 mg: 13  10 mg: 12  25 mg: 12  Placebo: 11 | 44.80 ± 11.43 | 61.82 ± 9.70 | 61.1% | 23.36 ± 3.19 | 16.72 ± 10.91 | 8.06 ± 0.57 | 9.98 ± 4.55 | 0.71 ± 0.19 | 0.30 ± 0.11 | 0.41 ± 0.17 | 114.08 ± 11.24 | 71.34 ± 12.08 | 89.86 ± 14.86 |
| Rosenstock 2018 (EASE-2) ^[^[^9^](#_ENREF_9)^]^ | Empagliflozin  10 mg: 243  25 mg: 241  Placebo: 239 | 45.17 ± 13.30 | 85.07 ± 17.77 | 46.75% | 29.17 ± 5.62 | 22.57 ± 12.65 | 8.10 ± 0.57 | 9.96 ± 3.57 | 0.71 ± 0.24 | 0.37 ± 0.16 | 0.35 ± 0.15 | 124.57 ± 15.35 | 76.20 ± 9.48 | 95.00 ± 18.46 |
| Rosenstock 2018 (EASE-2) ^[^[^9^](#_ENREF_9)^]^ | Empagliflozin  2.5 mg: 237  10 mg: 244  25 mg: 242  Placebo:238 | 43.05 ± 13.56 | 82.34 ± 16.95 | 49.64% | 28.23 ± 5.08 | 21.05 ± 12.05 | 8.18 ± 0.62 | 10.03 ± 3.56 | 0.71 ± 0.24 | 0.37 ± 0.14 | 0.35 ± 0.16 | 123.47 ± 14..96 | 75.56 ± 9.00 | 96.82 ± 19.69 |
| Kohei 2019A (2019) ^[^[^10^](#_ENREF_10)^]^ | Ipragliflozin  25 mg :9  50 mg :12  100mg :10  Placebo :10 | 44.16 ±  13.43 | 65.37 ± 8.73 | 29.27% | 24.76 ±  3.79 | 15.60 ± 10.11 | 8.61 ±  0.74 | 8.66 ±  3.68 | 0.69 ±  0.24 | 0.26 ± 0.10 | 0.43 ± 0.17 | NP | NP | 97.71 ± 24.60 |
| Kohei 2019B (2019) ^[^[^11^](#_ENREF_11)^]^ | Ipragliflozin  50 mg :12  Placebo :10 | 49.23 ±  12.9 | NP | 46.55% | 24.51 ± 2.91 | NP | 8.68 ± 0.80 | 10.73±  3.85 | 0.76 ±  0.36 | 0.47 ± 0.25 | 0.29 ± 0.15 | 93.79 ± 21.07 | NP | NP |
| Sands 2015 ^[^[^12^](#_ENREF_12)^]^ | Sotagliflozin  400mg: 16  Placebo: 17 | 39.58 (median) | 73.43  (median) | 48.48% | 27.64 ± 3.04 | 17.68  (median) | 7.96 ± 0.55 | 9.16 ± 3.67 | 0.60 | 0.32 | 0.28 | 118.98 ± 8.06 | NP | NP |
| Buse 2018 (inTandem 1) ^[^[^13^](#_ENREF_13)^]^ | Sotagliflozin  200mg:263  400mg: 262  Placebo: 268 | 46.06 ±  13.21 | 86.92 ±  18.07 | 48.17% | 29.66 ±  5.39 | 24.40 ±  12.80 | 7.57 ± 0.72 | 8.46 ±  3.63 | 0.75 ±  0.47 | 0.36 ± 0.27 | 0.40 ± 0.24 | 120.14 ±  14.35 | 76.04 ±  11.12 | 87.07 ± 19.18 |
| Danne 2018 (inTandem 2) ^[^[^14^](#_ENREF_14)^]^ | Sotagliflozin  200mg:261  400mg: 262  Placebo: 258 | 41.2 ± 13.44 | 81.66 ± 17.394 | 51.9% | 27.77 ± 5.121 | 18.40 ± 10.90 | 7.75 ± 0.835 | 9.06 ± 3.90 | 0.74 ± 0.280 | 0.36 ± 0.18 | 0.38 ± 0.24 | 123.1 ± 76.6 | 83.7 ± 32.1 | 92.03 ± 18.13 |
| Garg 2017 (inTandem 3) ^[^[^15^](#_ENREF_15)^]^ | Sotagliflozin  400mg: 699  Placebo: 703 | 42.85 ±  14.10 | 81.97 ± 17.08 | 49.71% | 28.19 ± 5.15 | 20.04 ± 12.25 | 8.23 ± 0.94 | 9.12 ± 3.91 | 0.70 ± 0.29 | 0.36 ± 0.19 | 0.34 ± 0.22 | 121.90 ± 15.05 | 76.55 ± 8.95 | 91.99 ± 20.85 |
| Baker 2019 (inTandem 4) ^[^[^16^](#_ENREF_16)^]^ | Sotagliflozin  75mg:35  200mg:35  400mg: 35  Placebo: 36 | 45.6 ± 13.3 | 85.5 ± 18.6 | 48.23% | 29.2 ± 5.6 | 24.1 ± 13.7 | 8.02 ± 0.83 | 8.74 ± 4.58 | 0.70 ± 0.32 | 0.31 ± 0.14 | 0.48 ± 0.27 | 119.6 + 14.80 | NP | 89.4 ± 17.7 |

### BMI, indicates body mass index; [eGFR, estimated glomerular filtration rate;](http://www.baidu.com/link?url=BQbNKaAd9dfHXdmKeouf5v5UmzGrP1a6R4yR6DDpCxB6ing1kYT3eHzl_nO7LATYHfGEyoMCC3UfdxjsHwmHkK) FPG, fasting plasma glucose; HbA1c, hemoglobin A1c; SBP, seated systolic blood pressure; DBP, seated diastolic blood pressure; NP not reported
